# Trial of Captain Moir

**Published:** 1831-01-01

**Authors:** 


### TRIAL OF CAPTAIN MOIR.

DR. VENABLES has put forth (in the Medical Gazette) a rather elaborate and sharp series of medico-legal commentaries on the late memorable trial of Captain Moir. It is but too notorious that, when medical men get entangled in forensic questions, they generally come out of court second best. Even if an individual happens to acquit himself with ability, it is almost certainly at the expense of some of his brethren, and consequently at the expense of the profession generally. Whenever a question of medical jurisprudence is mooted in a court of justice, each side may command as many opinions, at direct variance with each other, as they choose to issue subpœnas for—the result of which is, and must be, a degradation of the profession itself, in the eyes of the world. It is not with doctors as with lawyers :—the latter are paid for making black appear white; and their contradictory sentiments are looked for as efforts of ingenuity in the cause of their clients. But, when two medical men offer the most dissimilar evidence upon matters of science, the natural conclusion drawn by the public is, that there is no science, but mere conjecture in the case!

The rare example of unanimity among the medical witnesses, in Capt. Moir's trial, was owing to the circumstance that they were all on one side—that of the prosecution. Had the defendant's counsel called any, there would have been an exhibition of the usual conflicting testimony. Dr. Venables was in attendance, but not called. The series of strictures which he has published on the merits of the evidence and of the judgment of the court, shews that, had he had an opportunity, he would have endeavoured to impugn the one, and alter the other.

Dr. Venables condemns the treatment pursued in the case of Captain Moir's victim, and desires to shew that this maltreatment may have modified the result of the wound—that is, that it may have tended to the fatal trismus that caused the patient's death. The gist of the evidence produced by the chief professional witness, is contained in the following passage :—

"I found the deceased at Mrs. Baker's cottage, sitting by the fire. Upon examining him, I found that he was wounded in the right arm, which was much swollen both above and below the elbow-joint, but not at the joint itself. There was a wound on the inner side of the right arm, a little above the elbow, and also one in the opposite direction, a little above the olecranon, as if a ball had passed through; there was profuse hæmorrhage, but it had ceased. I called for some brandy, and gave the man a little, because he was exceedingly faint; I then applied a piece of linen to the wound, which bled a little while I was there. Before I left I put a tourniquet loosely on the arm, explaining the use of it to the attendant. *There was no bone fractured.*"

The remainder of the treatment we do not think worthy of much notice. The surgeon and those who were called in, found it necessary to bleed for the reduction of excitement—locked-jaw ensued—and the patient died. The main ground of censure taken up by Dr. Venables is the exhibition of brandy to a man who had lost a considerable quantity of blood after a gun-shot wound, but who was able to sit up by the fire. We do not mean to approve or even to sanction the procedure in question. It was apparently an unnecessary—perhaps an injudicious, or even injurious exhibition. That it may have accelerated and somewhat increased the subsequent excitement—and thus caused a few ounces more of blood to be abstracted for its reduction, is not improbable; but that it had any connexion with the trismus which ultimately supervened and destroyed life, we have neither facts nor reasons for supposing. Those who have seen gun-shot wounds, on a large scale, are well aware that tetanus is an accident which no human foresight can predict—and which no precautions can always prevent. It seems to depend much more on idiosyncrasy, climate, season, and circumstances over which we have no control, than on any particular mode of treatment pursued for the cure of the wound. Who then could conscientiously say, or rationally infer, that the exhibition of a little brandy to a man who was faint from loss of blood and agitation of mind consequent on a gun-shot wound, was the cause of the tetanus that supervened after the stage of excitement was over? In the course of the cross-examination, it came out indeed that the surgeon had administered some brandy when the wound was suppurating; and this is severely censured by Dr. Venables. The stimulant may not have been necessary, or even proper then, no more than in the first instance—but to connect this with the locked-jaw that followed, is, in our humble opinion, neither charitable nor just. We cannot, therefore, but think that Dr. V's censures are too severe, and too much in accordance with that general censorious spirit which now pervades the profession, and incites one medical man to worry another, whenever there is the least ground, real or imaginary, for criticism or blame. God forbid that we should endeavour to cloak ignorance, or defend malpractice in our profession; but it is a dangerous office to attribute mighty and fatal consequences to trifling deviations from rules of practice laid down in systematic works—one half of which, or more, are erroneous. Besides, it is almost impossible for people who are not on the spot, to judge of the actual indications which present themselves, from time to time, in any given case, and where the verbal descriptions are quite inadequate to an explanation of the practice which it is deemed necessary to pursue. But we shall leave this part of the subject.

In respect to the final sentence pronounced on Captain Moir by a jury of his countrymen, and approved by a wise and conscientious judge, we are led, upon mature reflexion, to believe that it was just and necessary. The act of firing at an unarmed and defenceless creature, like the deceased, without any personal struggle, and without any adequate provocation, was so savage and unmanly, that it deserved the utmost rigor of the law, had the consequence been the slightest wound of a finger. That the ball did not pass through the poor man's heart, instead of his arm, was no merit in Captain Moir, but only the result of chance; for it would be preposterous to maintain that the object was merely to carry away an arm. Captain Moir knew too well the uncertainty of a ball's course, to believe that he could unerringly hit a member and evade the trunk of a man at whom he fired. But even if he was perfectly confident in the perfection of his aim, he well knew that a man might die of hæmorrhage, in a field, without medical assistance; or sink under the effects of a gun-shot wound afterwards. In either case, the cruelty of the transaction called for condign punishment—infinitely more loud than that of counterfeiting a man's signature to put a few guineas in his purse. And supposing, for an instant, that the wounded man died from the ignorance of the surgeon who was called into attendance—that circumstance would not, in reason or in law, protect the inflictor of the wound from the full penalty of the final event of the case. Who put the deceased into the power of fire-arms first, and bad surgery (if such there was) afterwards? Captain Moir! We do not conceive that the death of the individual heightened the crime of the defendant, in this case; nor, that the mal-practice (if it had been proved) of the surgeon would have mitigated the crime of firing at an unarmed man, who was not even menacing the slightest degree of personal violence against the aggressor. The only plea which could have been put on the record, with any degree of success, would have been that of insanity. Although this plea was objected to by the prisoner, and abandoned as untenable by his counsel, we are strongly disposed to think that it was not quite unfounded in fact. It has been said that "ira furor brevis est." But we do not ground our conjecture on that. We can hardly think that any man in his sound senses would go out and *deliberately* fire at a man, against whom he had no real grounds of malice. The very argument which he adduced, was a proof of monomania—namely, that not only his house, but his fields were his castle; and that he might put any man to death who approached them without his permission!! No more insane idea ever entered the brain of an inmate of Bedlam.

